# To Explore the Molecular Mechanism of Acupuncture Alleviating Inflammation and Treating Obesity Based on Text Mining

**DOI:** 10.1155/2022/3133096

**Published:** 2022-09-05

**Authors:** YiKuan Du, LuLu He, XinNi Ye, ShuZhen Chen, GuanHao Li, YuanWei Yu, ErBai Ye, YiXing Huang, YuQi Zhou, WeiChui Zhang, Chun Yang

**Affiliations:** ^1^Central Laboratory, Affiliated Dongguan Hospital, Southern Medical University, Dongguan 523059, China; ^2^Dongguan Key Laboratory of Stem Cell and Regenerative Tissue Engineering, Guangdong Medical University, Dongguan 523808, China

## Abstract

**Objective:**

To explore the related mechanism of acupuncture affecting obesity by regulating inflammation using bioinformatics methods.

**Methods:**

The genes related to obesity, inflammation, and acupuncture and inflammation were mined using GenCLiP 3, and the intersecting genes were extracted using Venn diagram. The DAVID database was employed for pathway enrichment analysis and functional annotation of coexpressed genes. Then, the protein-protein interaction (PPI) network was constructed with the STRING database and visualized by the Cytoscape software and screened out important hub genes. Finally, the Boxplot and Survival Analysis of the hub genes in various cancers were performed by GEPIA.

**Results:**

755 genes related to obesity and inflammation and 38 genes related to acupuncture and inflammation were identified, and 24 coexpressed genes related to obesity, inflammation, and acupuncture were extracted from the Venn diagram. Eight hub genes including interleukin-6 (IL-6), interleukin-10 (IL-10), Toll-like receptor 4 (TLR4), signal transduction and transcriptional activation factor 3 (STAT3), C-X-C motif chemokine 10 (CXCL10), interleukin-17A (IL-17A), prostaglandin peroxide synthesis-2 (PTGS2), signal transistors, and transcriptional activation factor 6 (STAT6) were identified by gene ontology (GO), Kyoto Encyclopedia of Genes (KEGG), and PPI network analysis. Among them, IL-6 is suggested to play an essential role in the treatment of obesity and inflammation by acupuncture, and IL-6 was significant in both Boxplot and Survival Analysis of pancreatic cancer (PAAD). Therefore, in this study, the core gene, IL-6 was used as the breakthrough point to explore the possible mechanism of acupuncture in treating obesity and pancreatic cancer by regulating IL-6.

**Conclusion:**

(1) Acupuncture can regulate the expression of IL-6 through the TLR4/nuclear factor-*κ*B (NF-*κ*B) pathway, thereby alleviating inflammation, which can be used as a potential strategy for the treatment of obesity. (2) IL-6/STAT3 is closely related to the occurrence, development, and metastasis of pancreatic cancer. Acupuncture affecting pancreatic cancer through TLR4/NF-*κ*B/IL-6/STAT3 pathway may be a potential method for the treatment of pancreatic cancer.

## 1. Introduction

In recent years, the incidence of obesity has gradually increased in all groups and at all ages, making it one of the most serious public health and safety problems worldwide [1]. With the increase of various inflammatory factors in serum, such as C-reactive protein (CRP), IL-6, and tumor necrosis factor-*α* (TNF-*α*) [2], obesity is essentially a chronic low-grade inflammatory state that is difficult to eliminate. And this state can induce a variety of diseases, such as cardiovascular disease, insulin resistance, type II diabetes, hepatic steatosis, and cancer, making obesity an urgent problem to be solved.

At present, the common treatments for obesity include medication, surgery, Chinese medicine, acupuncture, and massage [3]. Compared with the side effects of medical treatment or the cost and risk of surgical treatment, acupuncture stands out as a green protocol. Various studies have shown that acupuncture may achieve the therapeutic purpose of obesity by promoting energy metabolism, reducing appetite, reducing intestinal energy absorption, alleviating inflammation, improving insulin signaling pathways, and other aspects [4]. Although many studies have proved that acupuncture is effective in treating obesity [5–7], its mechanism has not been clarified yet.

The amount of biomedical literature shows an exponential growth, which requires a large quantity of time for researchers to fully read the existing relevant literature. In addition, the diversity of target genes in traditional Chinese medicine makes it inefficient to blindly verify the pathways one by one through experimental methods. Text mining technology is a branch of data mining technology, but compared with other data mining methods, text mining increases the sensitivity of a search by identifying more words, word forms, or phrases and thus broadens the range of studies including related records [8]. The goal of text mining is to summarize and extract the most relevant information from a large number of publications by applying algorithms and statistical methods and establish a relationship between different studies, so that researchers can more effectively obtain the required information from a large number of literature [9], and moreover, research results are usually presented as structured data. With the development of natural language processing (NLP) technology, extracting valuable information from biomedical literature has been increasingly welcomed by researchers [10]. Applying the text mining method to the exploration of disease treatment pathways is to obtain relevant verified gene information from published articles, construct a candidate gene list for disease treatment through algorithms and deep learning processes, and put forward the pathway hypothesis through further computer analysis. Text mining has become an important and efficient method for exploring key genes and related pathways of diseases. Unfortunately, at present, there are still few studies applying text mining technology to explore the mechanism of acupuncture treatment for obesity.

In this study, we used GenCLiP 3 to deeply mine the key genes and pathways related to the occurrence and development of obesity and further screen through gene ontology analysis, pathway enrichment annotation, and protein-protein interaction analysis, in an attempt to find out the key targets and related pathways of acupuncture therapy for obesity, providing a certain reference for subsequent related research and the exploration of therapeutic methods.

## 2. Materials and Methods

### 2.1. Obtain Coexpressed Genes

GenCLiP 3 (http://ci.smu.edu.cn/genclip3/analysis.php) is a web-based text mining server that, like other text mining tools, focuses on how genes relate to various topics, such as various biological processes and diseases, by extracting gene-related information from the PubMed literature. The difference is that GenCLiP 3 has established a new model, which integrates Sphinx and MySQL to establish the index of literature and its corresponding genes, and identifies the gene-keyword correlation by comprehensively considering the cooccurrence frequency of genes and keywords to be retrieved in the sentences and abstracts of the literature [11], with the characteristics of good accuracy and strong real-time. Efficient use of the GenCLiP 3 website will allow us to quickly retrieve functionally relevant genes with complex and customized search conditions from a large body of literature.

In this study, phenotypic and protein (gene) retrieval was set up on the website of GenCLiP 3, with “(obesity' OR ‘adiposis' OR ‘corpulency') AND (Inflammation)” and “(‘acupuncture' OR ‘needling' OR ‘prodding') AND (Inflammation)” as search terms, respectively, to obtain the information of two groups of genes. Then, by intersecting the two groups of genes using a Venn diagram (https://bioinfogp.cnb.csic.es/tools/venny/), the coexpressed genes related to obesity, inflammation, and acupuncture were obtained for subsequent analysis.

### 2.2. GO and KEGG Enrichment Analysis

To understand the biological functional pattern of coexpressed genes, GO function and KEGG pathway analysis of the selected coexpressed genes were performed using the online analysis website of DAVID (version 6.8). The first 15 results were independently extracted, and the bubble diagram was drawn with the ggplot2 software package in R language. *P* < 0.05 was considered statistically significant.

### 2.3. Construction of the PPI Network and Screening of Key Genes

To further explore the potential relationship between coexpressed genes, we first imported the coexpressed genes into the “Multiple proteins” option of the STRING online website (version 11.0b) and constructed the initial PPI network. Subsequently, we imported the initial PPI network into the Cytoscape software to construct a visual network model for further analysis.

Hub genes are often important action targets and focus in the research of biological processes. Next, we screened hub genes according to the network topology through the PPI network. The important clustering functional modules were screened in Cytoscape software using the MCODE plug-in so that we could observe the subnetworks where the coexpressed genes interacted most closely in the dimension of protein. Since the PPI network was constructed through Cytoscape, more nodes and edges tended to mask the true hub genes and affected the screening process. Therefore, we used the CytoHubba plug-in to predict and explore key nodes and subnetworks in a given network. In the Hubba node option of the CytoHubba plug-in, two network topology algorithms, Degree and The Maximum Clique Centre (MCC), were selected to calculate and analyze the PPI network structure and the connection weights between nodes, and then, the respective top ten genes were sequenced. In order to synthesize the important nodes in the biological network, we selected the intersection of the top ten genes screened out by the two algorithms as the final hub genes.

### 2.4. Prognosis Analysis of the Hub Genes in Various Cancers

In view of the close relationship between obesity and cancer, we next observed the correlations between hub genes and various types of cancer on the GEPIA website (http://gepia.cancer-pku.cn) using the published gene sequencing information. GEPIA is an interactive web server that integrates information from gene expression datasets from TCGA and GTEx common databases to help us perform comprehensive and complex data mining tasks [12]. We placed the screened hub genes on the GEPIA website and analyzed the expression level of hub genes in various cancers using the Boxplot tool, which was conducive to the discovery of cancer-specific differentially expressed genes (DEGs). The overall survival curves of screening DEGs were generated using the Survival Analysis tool in the GEPIA website, and we selected median as the group cutoff for survival plots.

## 3. Results

### 3.1. Acquisition of Coexpressed Genes

**Figure 1:**
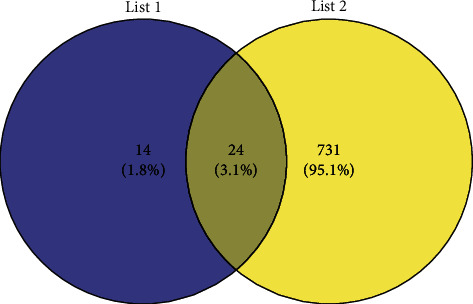
Coexpressed genes of acupuncture, obesity, and inflammation. List 1 contains 38 genes related to acupuncture and inflammation, and list 2 contains 755 genes related to obesity and inflammation. The overlapping parts of list 1 and list 2 are coexpressed genes.

We retrieved 755 genes related to obesity and inflammation and 38 genes related to acupuncture and inflammation on the GenCLiP 3 website according to the method shown in Section 2.1. Using the Venn diagram to obtain the intersection, we obtained the 24 coexpressed genes, which were mentioned most frequently in articles related to obesity, inflammation, and acupuncture, namely, IL-10, proopiomelanocortin (POMC), mitogen-activated protein kinase (MAPK14), CRP, brain-derived neurotrophic factor (BDNF), interleukin 1 receptor-like 1 (IL1RL1), interleukin 13 (IL-13), Janus kinase 2 (JAK2), STAT3, myeloid differentiation factor 88 (MYD88), RAC-alpha serine/threonine-protein kinase (AKT1), fibronectin type III domain-containing protein 5 (FNDC5), TLR4, IL-6, STAT6, B-cell lymphoma 2 (BCL2), IL-17A, CXCL10, C-C Motif Chemokine Ligand 11 (CCL11), Superoxide Dismutase 1 (SOD1), PTGS2, cholecystokinin (CCK), Oxytocin (OXT), and thioredoxin-interacting protein (TXNIP), as shown in Figure 1.

### 3.2. Enrichment Analysis and Functional Annotation of Coexpressed Genes

**Figure 2:**
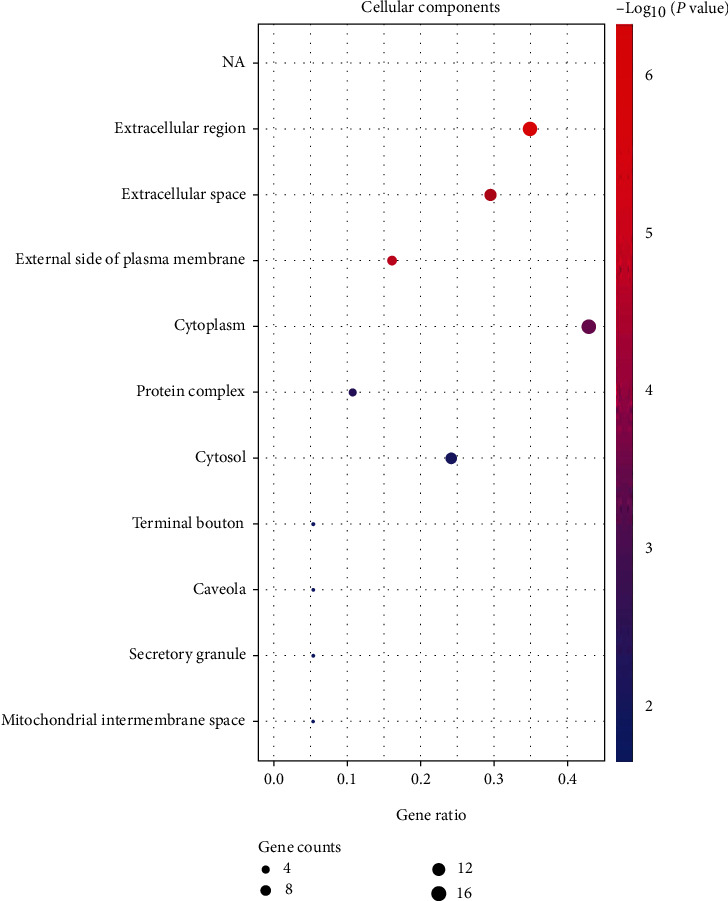
Bubble diagram of cell component enrichment analysis of coexpressed genes. The dot size represents the counts of overlapped genes between the coexpressed gene list and the total gene list of the given GO term. Gene ratio represents the counts of overlapped genes for each GO item divided by the total number of genes in the coexpressed gene list. The color scale represents the value of –log10 (*P* value).

**Figure 3:**
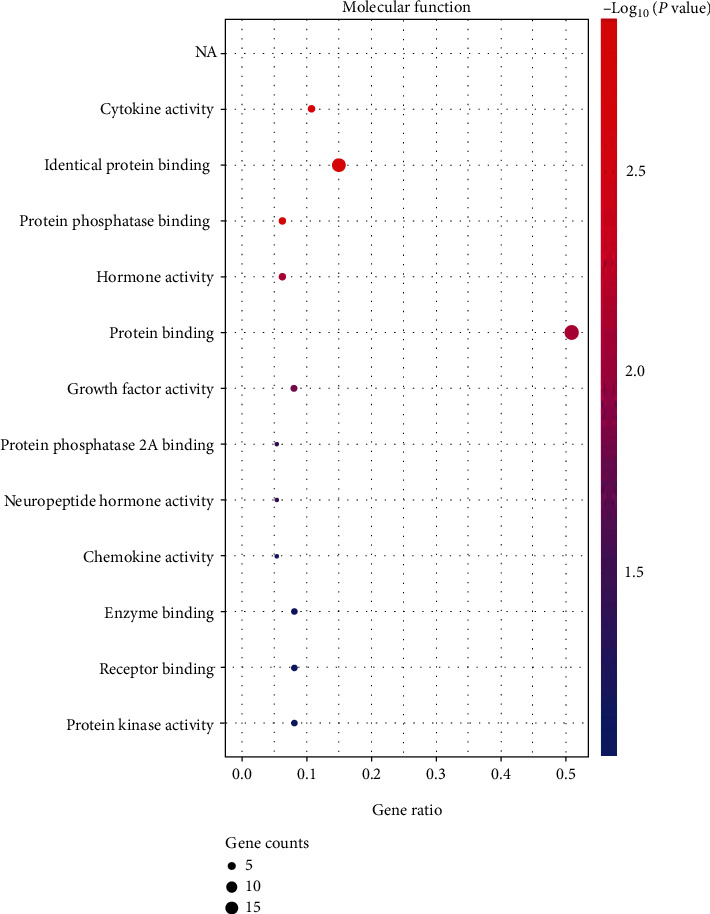
Bubble diagram of molecular function enrichment analysis of coexpressed genes. The dot size represents the counts of overlapped genes between the coexpressed gene list and the total gene list of the given GO term. Gene ratio represents the counts of overlapped genes for each GO item divided by the total number of genes in the coexpressed gene list. The color scale represents the value of –log10 (*P* value).

**Figure 4:**
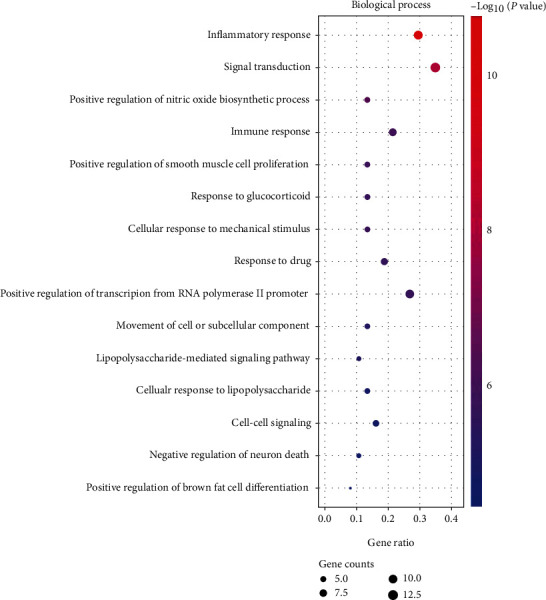
Bubble diagram of biological process enrichment analysis of coexpressed genes. The dot size represents the counts of overlapped genes between the coexpressed gene list and the total gene list of the given GO term. Gene ratio represents the counts of overlapped genes for each GO item divided by the total number of genes in the coexpressed gene list. The color scale represents the value of –log10 (*P* value).

We used the DAVID database to reveal more specific functional patterns of coexpressed genes. Among them, GO analysis showed that the cellular components (CC) of most proteins related to obesity, inflammation, and acupuncture were mainly located in intracellular and extracellular compartments. The intracellular compartments included cytoplasm, cytosol, caveola, and mitochondrial intermembrane space, while the extracellular compartments included extracellular region, extracellular space, and external side of plasma membrane, as shown in Figure 2. Molecular function (MF) analysis showed that most proteins related to obesity, inflammation, and acupuncture had the functions of protein binding, identical protein binding, cytokine activity, etc., as shown in Figure 3. Biological process (BP) analysis showed that most genes related to obesity, inflammation, and acupuncture were mainly involved in signal transduction, inflammatory response, positive regulation of transcription from RNA polymerase II promoter, immune response, response to drug, etc., as shown in Figure 4.

**Figure 5:**
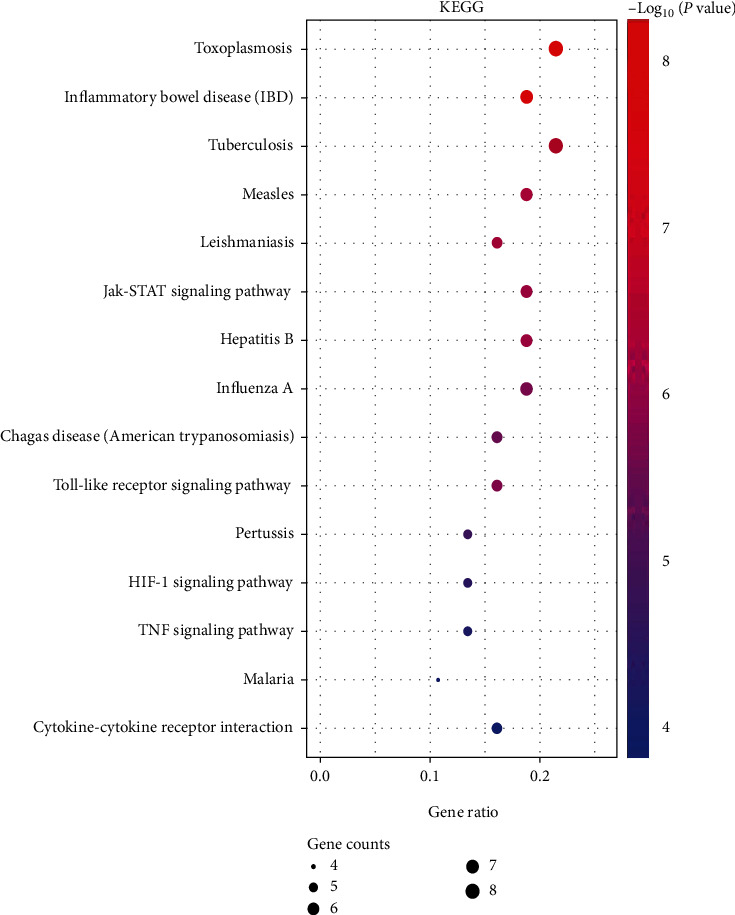
KEGG pathway enrichment analysis of coexpressed genes. The dot size represents the counts of overlapped genes between the coexpressed gene list and the total gene list of the given KEGG term. Gene ratio represents the counts of overlapped genes for each KEGG item divided by the total number of genes in the coexpressed gene list. The color scale represents the value of –log10 (*P* value).

To elucidate the pathways of coexpressed genes involved in obesity, inflammation, and acupuncture, we performed enrichment analysis of the KEGG pathway, where these genes are mainly enriched in four subcategories: signal transduction pathway, parasitic infection-related pathway, bacterial infection-related pathway, and viral infection-related pathway. Signal transduction pathways include Jak-STAT signaling pathway, Toll-like receptor signaling pathway, HIF-1 signaling pathway, tumor necrosis factor signaling pathway, and cytokine-cytokine receptor interaction. The parasitic infection-related pathways include toxoplasmosis, chagas disease (American trypanosomiasis), and malaria. The bacterial infection-related pathways include inflammatory bowel disease, tuberculosis, leishmaniasis, and pertussis. The viral infection-related pathways include measles, as shown in Figure 5 and Table 1.

### 3.3. PPI Network Construction and Screening of Key Genes

**Figure 6:**
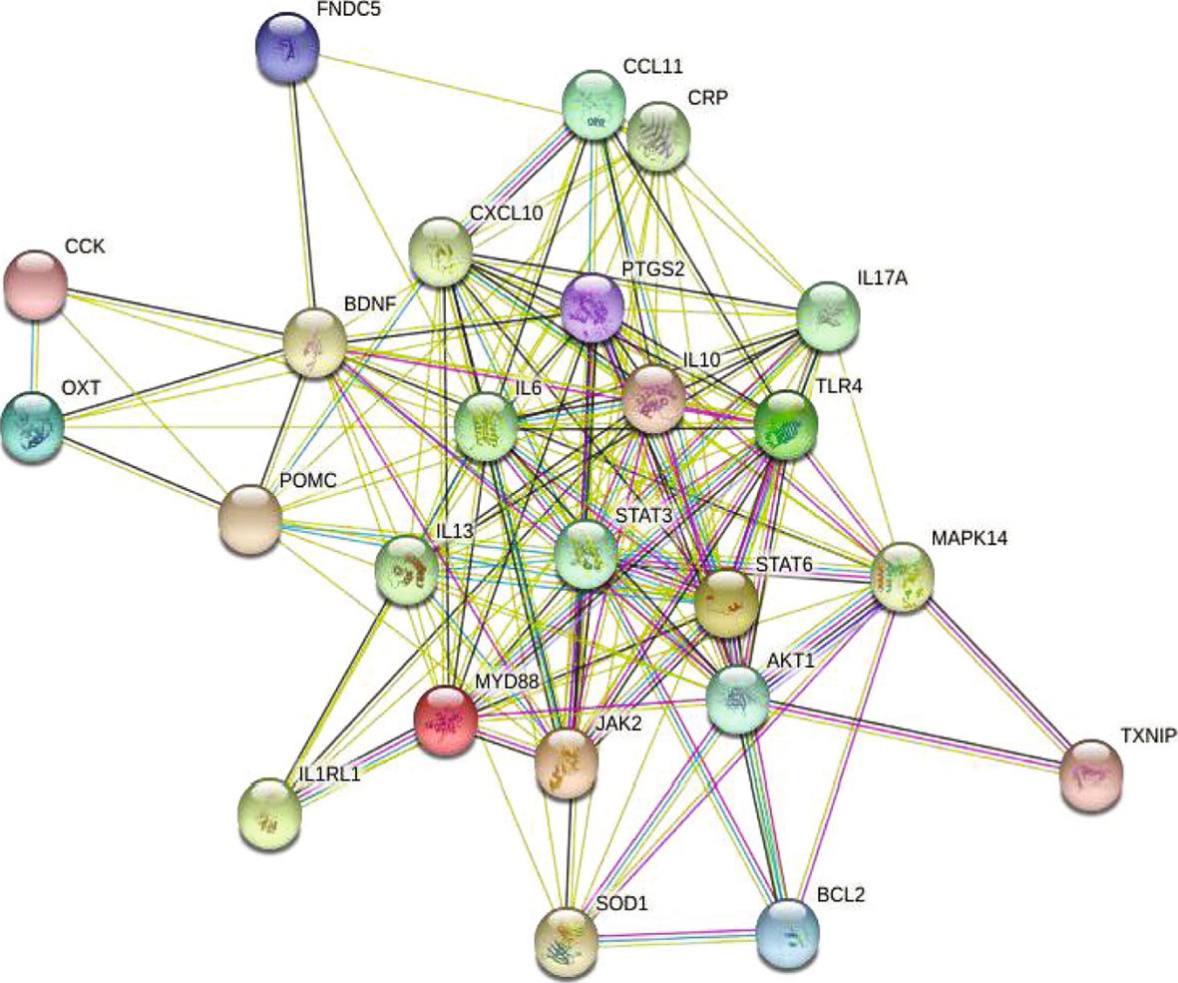
The PPI network nodes of 24 target genes obtained by intersection represent proteins, and different colors represent different categories of proteins. The thickness of the connection between nodes represents the degree of data in support of their interaction relationship, and the thicker the connection line, the more data it supports.

In order to clarify the tissue-specific interaction between coexpressed genes, a specific protein-protein interaction network was established. The results of the initial PPI network established on the STRING site are shown in Figure 6. The network consists of 24 protein nodes and 142 protein-protein interaction chains.

**Figure 7:**
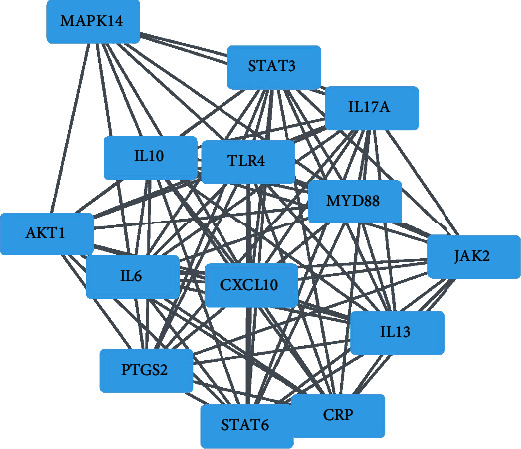
Ranking of protein-protein interactions of the top 14 target genes of MCODE. Network nodes represent proteins, while edges represent associations of proteins.

**Figure 8:**
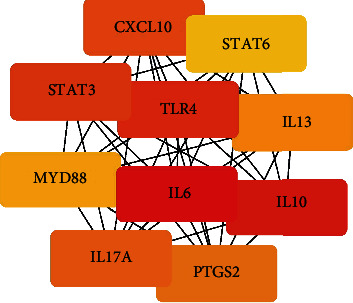
The key gene interaction network with MCC ranked in the top 10. Edges represent the protein-protein associations. The redder the color is, the higher the MCC scores will be. And a higher score indicates more importance.

**Figure 9:**
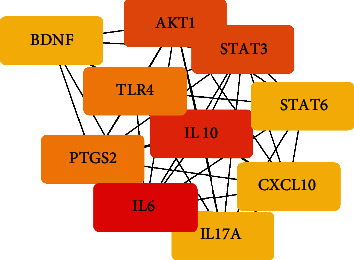
The key gene interaction network ranked in the top 10 by Degree. The redder the color is, the higher the Degree scores will be. And a higher score indicates more importance.

**Figure 10:**
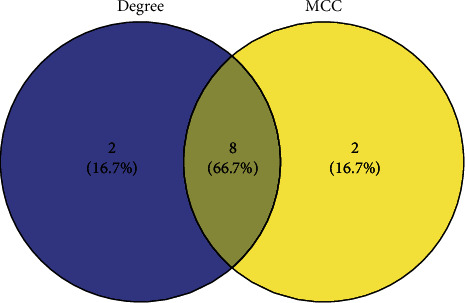
The intersection of the top 10 genes with Degree and MCC algorithms.

To study the specific hub gene related to obesity, inflammation, and acupuncture, we imported the initial PPI network into the Cytoscape software for visual analysis. The important clustering functional modules obtained by the MCODE plug-in filtering include 14 nodes and 84 edges, as shown in Figure 7, where the filtering conditions are Degree Cutoff = 2, Node Score Cutoff = 0.2, K − Core = 2, and *Max* Depth = 100. The top 10 hub genes screened by MCC method in the CytoHubba plug-in were as follows: IL-6, IL-10, TLR4, STAT3, CXCL10, IL-17A, PTGS2, IL-13, MYD88, and STAT6, as shown in Figure 8. The top 10 hub genes screened by Degree method were as follows: IL-6, IL-10, AKT1, STAT3, PTGS2, TLR4, BDNF, STAT6, CXCL10, and IL-17A, as shown in Figure 9. Taking the intersection of hub genes obtained by Degree and MCC methods, a total of eight common hub genes were obtained as the finally screened hub genes, as shown in Figure 10.

### 3.4. Analysis of Expression Differences and Prognosis of the Hub Genes

**Figure 11:**
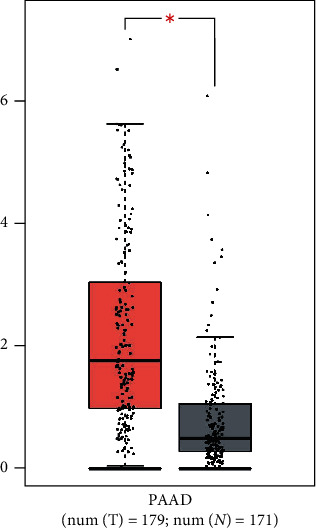
Expression differences of IL-6 in pancreatic cancer and normal pancreatic tissues in the GEPIA database. Red for tumor tissue (T) and gray for normal tissue (N): *P* < 0.05.

**Figure 12:**
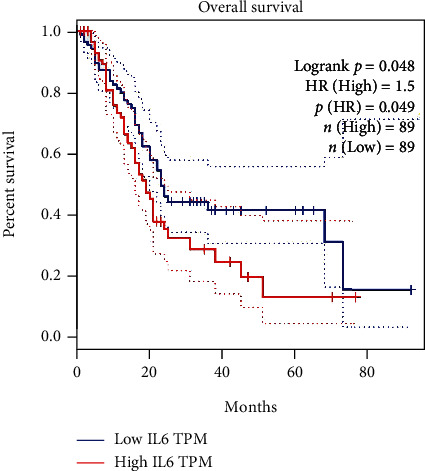
Effect of IL-6 expression level on the overall survival of PAAD patients in the GEPIA database. Patients were divided into low- and high-expression groups according to median value. Both low- and high-expression groups contain 89 patients with PAAD.

After screening by the Boxplot tool on the GEPIA website, in the list of 24 coexpressed genes, genes which showed significantly differential expression (with a *P* value < 0.05) and with significance in prognosis analysis, in various cancers, were as follows: AKT1, BCL2, CCK, CCL11, CXCL10, IL1RL1, IL6, JAK2, MAPK14, MYD88, PTGS2, SOD1, STAT3, STAT6, TLR4, and TXNIP. The expression level of IL-6 in PAAD was significantly higher than in normal pancreatic tissues, as shown in Figure 11. Furthermore, survival analysis showed that the survival rate was significantly lower in patients with a high IL-6 expression level than in those with a low IL-6 expression level, with a *P* value of 0.048, as shown in Figure 12.

## 4. Discussion

Obesity is considered a chronic low-level systemic inflammation that predisposes to diseases such as diabetes, cardiovascular disease, and malignancy. Obesity has seriously damaged people's health and quality of life. With the continuous exploration of the treatment of obesity, more and more literature support that, as an effective means to alleviate inflammation, acupuncture can influence and treat obesity by regulating the expression of inflammatory factors and their related pathways. However, we still need to clarify as much as possible why acupuncture therapy is useful and what are the key targets and molecular mechanisms of the treatment process. Only by solving these problems can we provide more solid and powerful molecular biological evidence for acupuncture treatment of obesity and provide new ideas for the treatment of obesity. In this study, we performed text mining on the published articles related to obesity, inflammation, and acupuncture, established a collection of coexpressed genes related to our research topic, and analyzed their biological functions, pathways, and PPI network. We identified a total of eight important hub genes. Among them, we found that IL-6 and its related pathways play an important role in treating obesity through alleviating inflammation. Therefore, we selected IL-6, which is highly relevant among the hub genes screened, as an entry point to uncover the mechanism by which acupuncture regulates inflammation to affect obesity and suggested the importance of IL-6 in the prognostic of PAAD.

As an inflammatory factor associated with obesity, the level of IL-6 is positively correlated with obesity [13, 14]. In obese people, the enlarged adipocytes in adipose tissue, altered mitochondria, local hypoxia-induced oxidative stress, and endoplasmic reticulum stress can promote the secretion of proinflammatory cytokines, such as IL-6 and TNF-*α*, and the secretion of IL-6 is positively correlated with adipocyte size [15, 16]. In addition, during inflammation, there is also a positive feedback effect of calmodulin 0 activating TLR4 and downstream inflammatory signaling pathways to promote IL-6 release [17]. At the level of gene expression regulation, Meta data analysis showed that the -174G>C polymorphism in the promoter region of IL-6 was associated with obesity [18]. In obese states, lipolysis is enhanced in vivo, and high concentrations of free fatty acids can upregulate the expression of Krüppel-like factor 7 (KLF7) via TLR4, and KLF7 acts as a transcription factor directly binding to the IL-6 DNA promoter region at 5′-1696~13863′bp and 5′-354~1043′bp sequences to activate IL-6 transcription [19]. There is no doubt about the increase of serum IL-6 in obese people. At the same time, IL-6 will in turn affect obesity, but its mechanism is relatively complex. IL-6 is a typical adipokine, with 15-35% of serum IL-6 coming from adipose tissue [20]. The receptor for IL-6 (IL-6R) is expressed only in hepatocytes, epithelial cells, and leukocytes, but IL-6 can act on all cells via a trans-signaling pathway [21, 22]. The role of IL-6 in obesity is distinct in the central nervous system and in peripheral tissues [23]. Intraventricular injection of low doses of IL-6 increases energy expenditure and has some antiobesity effect, whereas intraperitoneal injection does not have the same effect [24]. The role of IL-6 in obesity-related energy regulation is also controversial. On the one hand, elevated systemic levels of IL-6 activate STAT3 and subsequently AMP-activated protein kinase (AMPK), leading to impaired insulin signaling and inhibition of energy metabolism [25]. On the other hand, there are new evidences that IL-6 inhibits lipogenesis by activating STAT3-mediated downregulation of peroxisome proliferator-activated receptor (PPAR*γ*) [26]. When IL-6 is elevated and STAT3 is activated in obese patients, it may be a new therapeutic strategy if it inhibits the AMPK pathway and promotes the inhibitory effect on PPAR*γ*, thereby activating part of the pathway that inhibits lipogenesis. IL-6-deficient mice will be obese due to reduced energy consumption, suggesting that IL-6 can enhance energy metabolism to prevent obesity and insulin resistance [25]. Although there are many controversial studies on the effects of IL-6 on obesity, IL-6 must be the key in the process of energy metabolism and adipogenesis, which may involve pathways like IL-6/STAT3/AMPK and IL-6/STAT3/PPAR*γ*. Meta-analysis suggests that the IL-6 pathway is involved in body weight regulation and that regulation of IL-6 signaling may be a potential therapeutic pathway [25]. Therefore, we believe that although there are numerous and intricate mechanisms involved in the regulation of obesity by IL-6, IL-6 could be a potential target for the treatment of obesity.

Acupuncture plays a therapeutic role in systemic inflammation and various related diseases. Electroacupuncture (EA) regulates downstream effectors of the TLR4/NF-*κ*B signaling pathway, such as reducing the secretion of inflammatory factors such as TNF-*α*, interleukin-1 (IL-1), and IL-6 and effectively reducing pneumonia [27] and brain ischemic inflammatory response [28], and it plays a neuroprotective role in ischemic stroke [29]. The TLR4/NF-*κ*B pathway may also have a significant impact on acupuncture treatment of obesity through IL-6 [30]. TLR4/NF-*κ*B is an important pathway that regulates IL-6. In the mechanistic study of the drug, modified Zhujing formula [31], Elephantopus scaber Linn [32] decreased the expression of IL-6 by inhibiting TLR4 and downregulating NF-*κ*B. In atherosclerosis (AS) clinical drug trials, SQMXTJN blocked TLR4/NF-*κ*B signaling pathway and downregulated the expression of TLR4, NF-*κ*B, IL-6, TNF-*α*, and other proteins in the serum of AS patients, thereby reducing inflammation, acting as an antiatherosclerotic agent and slowing down the progression of AS [33]. In addition, acupuncture may act on miRNA [34], such as upregulating the expression of miR-30b-5p, and then inhibiting the TLR4/NF-*κ*B signaling pathway [35]. EA treatment of Zusanli (ST36) showed that by enhancing the expression of cannabinoid receptor 2 (CB2R), it inhibited Ca^2+^ inward flow, leading to inactivation of TLR4/NF-*κ*B signaling and decreased the expression of IL-6 [36]; EA downregulates the expression of high mobility group protein 1 (HMGB1), which activates TLR4 by binding to the small secreted glycoprotein MD-2 [37]. Thus, acupuncture may affect the regulation of obesity by inhibiting the activation of TLR4 via HMGB1 and thus downregulating NF-*κ*B/IL-6 pathway. It has also been conclusively shown that acupuncture can reduce the activity of NF-*κ*B p65 by modulating intestinal TLR4 and then inhibiting its interaction with NF-*κ*B p65 [38]. Acupuncture indirectly affects TLR4 through its effects on miRNA, CB2R, and HMGB1 or directly by modulating TLR4 and thus directly inhibiting the TLR4/NF-*κ*B signaling pathway and downregulating IL-6 expression. The downregulation of IL-6 and other inflammation-related factors by acupuncture is important mechanism for the therapeutic effects of acupuncture on systemic inflammation and various related diseases. As mentioned above, IL-6 is a very promising therapeutic target for obesity, and acupuncture is likely to modulate inflammation and thus affect obesity through the TLR4/NF-*κ*B/IL-6 signaling pathway.

Numerous studies have shown that obesity is closely related to PAAD [39]. We analyzed genetic data from pancreatic cancer and found that IL-6 expression was much higher in PAAD than in normal pancreatic tissue. Also, PAAD patients with high levels of IL-6 expression had significantly lower survival rates (*P* value 0.048). Therefore, we speculated whether acupuncture can be used to play a certain auxiliary role in the treatment of PAAD by targeting IL-6. PAAD is one of the most aggressive and lethal cancer types, and patients have extremely poor prognosis and low survival rate due to no obvious symptoms in the early stage, rapid disease progression, and low efficiency of chemotherapy, while the incidence of pancreatic cancer is increasing year by year, which seriously threatens people's life and health [40]. Therefore, it is especially important to explore the pathological mechanism and find the corresponding treatment protocols. As a major member of the tumor inflammatory microenvironment, the proinflammatory cytokine IL-6 is involved in the development and progression of pancreatic cancer [41, 42]. The IL-6/STAT3 signaling pathway has been proposed as a major linking mechanism between the inflammatory tumor microenvironment and pancreatic cancer [43, 44], which may promote the initiation and progression of tumors by regulating oncogenes [45] and epigenetic modifications of tumor suppressor genes [46, 47]. In contrast, inhibition of the IL-6 signaling pathway significantly reduces primary tumor growth and recurrence in an orthotopic xenograft pancreatic cancer model. The suppressor of cytokine signaling 3 (SOCS3) has been proven to inhibit cancer in breast cancer [48], hepatocellular carcinoma [49], small-cell lung cancer [50], prostate cancer [51], and other tumors, while IL-6/STAT3 signaling can induce SOCS3 methylation through DNA methyltransferase 1 (DNMT1) [47], which may promote the growth and metastasis of pancreatic cancer in vitro and in vivo. Overexpression of neurofibrillary protein-1 (NRP-1) has been shown to be correlated with the invasive behavior of tumor cells [52]. Related studies suggest that NRP-1 may be regulated by STAT3, and that NRP-1 is upregulated in pancreatic neuroendocrine tumor tissues and correlates with the metastatic ability of pancreatic neuroendocrine tumor cells [53]. Regenerative gene protein (REG3A) overexpression is associated with excessive proliferation, invasion, migration, distant metastasis, and tumor aggressiveness [54, 55]. Relevant studies have shown that REG3A acts as a growth-promoting cell regulator and interacts with JAK2/STAT3 signaling to form a positive feedback loop to accelerate pancreatic cancer cell growth under IL-6-related inflammatory conditions [56]. In addition, acupuncture has been used clinically for the treatment of cancer pain, and numerous publications have demonstrated the effectiveness of acupuncture in the treatment of cancer-caused fatigue [57]. This therapeutic effect of acupuncture may be associated with the reduction of inflammatory cytokines like IL-6 [58]. Therefore, it is reasonable to believe that IL-6 could also be a potential target for the prevention and adjuvant therapy of pancreatic cancer. Targeting IL-6 in acupuncture treatment of pancreatic cancer is a promising research direction, which may involve IL-6/STAT3 axis, DNMT1-induced SOCS3 methylation, NRP-1, etc.

In this study, making full use of the intelligent NLP-based text mining technology can help us quickly find the genes that play a key role in the treatment of obesity and inflammation by acupuncture from the vast amount of literature information and use them for subsequent analysis. Text mining can not only keep up with the latest research results but also be a powerful tool for us to generate new hypotheses. However, since the results obtained in this research are based on the prediction of text mining and considering the automation in the literature retrieval process and the possibly hidden false-positive results in the literature [59], as well as some valuable genes to be found that are not published in the text form may be ignored in the obtained coexpressed gene results in this research, thus, our research results need further experimental exploration and verification.

## 5. Conclusions

To date, although there are quantities of clinical studies on acupuncture for the treatment of obesity, there are still few basic studies on its specific pathways, especially inflammatory pathways. However, our results provide new insights into the pathogenesis of obesity and the possible molecular mechanisms of acupuncture in the treatment of obesity and inflammation. This study found 24 coexpressed genes related to obesity, inflammation, and acupuncture based on text mining. By analyzing the GO, KEGG, and PPI networks of the genes, we found that the core gene IL-6 and TLR4/NF-*κ*B/IL-6 pathway are associated with acupuncture affecting obesity by regulating inflammation, further revealing the importance of IL-6/STAT3 and its downstream pathway in pancreatic cancer and providing some theoretical basis for adjuvant treatment of pancreatic cancer targeting IL-6. However, in this study, we have not yet delved into the interactions of other core genes in the mechanism of acupuncture regulating inflammation and affecting obesity, but this could be a direction for subsequent research.

## Figures and Tables

**Table 1:** Enrichment analysis of the top 15 KEGG pathway gene sets.

Entry	Process	*P* value	Genes
hsa05145	Toxoplasmosis	6.07*E*-09	IL-10, STAT3, BCL2, AKT1, JAK2, MAPK14, TLR4, MYD88
hsa05321	Inflammatory bowel disease	8.69*E*-09	IL-10, IL-6, STAT3, IL-13, STAT6, TLR4, IL-17
hsa05152	Tuberculosis	1.66*E*-07	IL-10, IL-6, BCL2, AKT1, JAK2, MAPK14, TLR4, MYD88
hsa05162	Measles	7.16*E*-07	IL-6, STAT3, IL-13, AKT1, JAK2, TLR4, MYD88
hsa05140	Leishmaniasis	7.84*E*-07	IL-10, JAK2, MAPK14, PTGS2, TLR4, MYD88
hsa04630	Jak-STAT signaling pathway	1.19*E*-06	IL-10, IL-6, STAT3, IL-13, AKT1, STAT6, JAK2
hsa05161	Hepatitis B	1.19*E*-06	IL-6, STAT3, BCL2, AKT1, STAT6, TLR4, MYD88
hsa05164	Influenza A	3.47*E*-06	CXCL10, IL-6, AKT1, JAK2, MAPK14, TLR4, MYD88
hsa05142	Chagas disease (American trypanosomiasis)	5.26*E*-06	IL-10, IL-6, AKT1, MAPK14, TLR4, MYD88
hsa04620	Toll-like receptor signaling pathway	5.77*E*-06	CXCL10, IL-6, AKT1, MAPK14, TLR4, MYD88
hsa05133	Pertussis	3.55*E*-05	IL-10, IL-6, MAPK14, TLR4, MYD88
hsa04066	HIF-1 signaling pathway	9.38*E*-05	IL-6, STAT3, BCL2, AKT1, TLR4
hsa04668	TNF signaling pathway	1.43*E*-04	CXCL10, IL-6, AKT1, MAPK14, PTGS2
hsa05144	Malaria	2.57*E*-04	IL-10, 1L-6, TLR4, MYD88
hsa04060	Cytokine-cytokine receptor interaction	3.10*E*-04	IL-10, CXCL10, IL-6, CCLII, IL-13, IL-17A

Entry: accession number from the KEGG PATHWAY database.

## Data Availability

The data used to support the findings of this study are included within the article.
